# Dissimilar Deformation of Fluid- and Gel-Phase Liposomes upon Multivalent Interaction with Cell Membrane Mimics Revealed Using Dual-Wavelength Surface Plasmon Resonance

**DOI:** 10.1021/acs.langmuir.1c03096

**Published:** 2022-02-14

**Authors:** Karin Norling, Mattias Sjöberg, Marta Bally, Vladimir P. Zhdanov, Nagma Parveen, Fredrik Höök

**Affiliations:** † Division of Nano and Biophysics, Department of Physics, Chalmers University of Technology, 412 96 Gothenburg, Sweden; ‡ Department of Clinical Microbiology, Umeå University, 901 85 Umeå, Sweden; § Wallenberg Centre for Molecular Medicine, Umeå University, 901 85 Umeå, Sweden; ∥ Boreskov Institute of Catalysis, Russian Academy of Sciences, Novosibirsk 630090, Russia

## Abstract

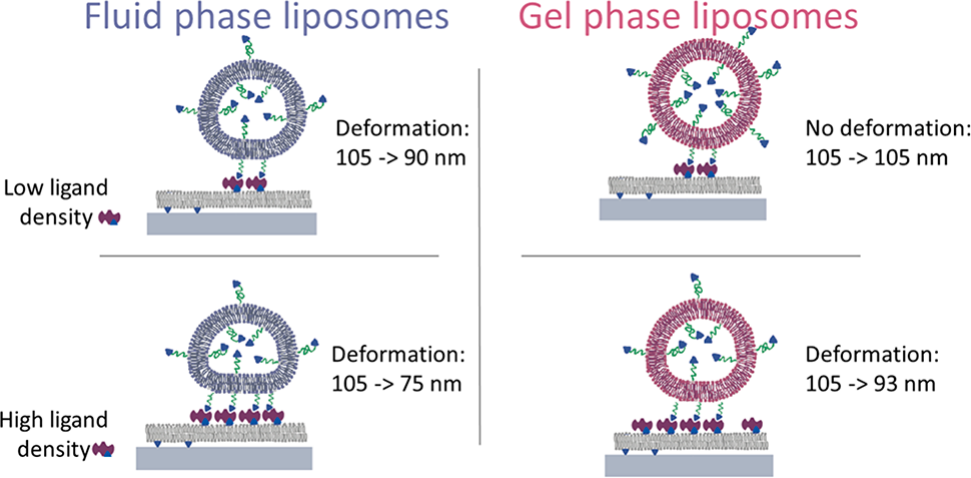

The mechanical properties of biological nanoparticles play a crucial role in their interaction with the cellular membrane, in particular for cellular uptake. This has significant implications for the design of pharmaceutical carrier particles. In this context, liposomes have become increasingly popular, among other reasons due to their customizability and easily varied physicochemical properties. With currently available methods, it is, however, not trivial to characterize the mechanical properties of nanoscopic liposomes especially with respect to the level of deformation induced upon their ligand–receptor-mediated interaction with laterally fluid cellular membranes. Here, we utilize the sensitivity of dual-wavelength surface plasmon resonance to probe the size and shape of bound liposomes (∼100 nm in diameter) as a means to quantify receptor-induced deformation during their interaction with a supported cell membrane mimic. By comparing biotinylated liposomes in gel and fluid phases, we demonstrate that fluid-phase liposomes are more prone to deformation than their gel-phase counterparts upon binding to the cell membrane mimic and that, as expected, the degree of deformation depends on the number of ligand–receptor pairs that are engaged in the multivalent binding.

## Introduction

The interaction of nanosize particles (∼100 nm in diameter) with cellular lipid membranes plays an important role in vivo (classical examples are virions^1^ and extracellular vesicles or, in other words, liposomes^2^) and also in the context of the development of new generations of drug and vaccine delivery vehicles, including lipid nanoparticles^3^ as well as liposomes and micelles.^4^ This interaction is typically multivalent, i.e., mediated by a large number of weak bonds, and strongly depends on the mechanical properties of the involved nanoparticles, the cellular membrane, and its associated cytoskeleton.^4,5^ The theory predicts that in the case of rigid nanoparticles the interplay of these factors results in the existence of an optimal nanoparticle size (∼100 nm) for penetration through a cellular membrane.^6^ In the case of nanoparticles prone to deformation (e.g., liposomes), the prediction is that the ligand–receptor interactions required for their full wrapping by the cell membrane may not be sufficient to overcome the bending energy of the cell membrane, which can in turn result in partial membrane wrapping and trapping of nanoparticles on the cell surface.^7^ Experimentally, there are, however, conflicting results regarding the significance of particle rigidity: for immune cells and endothelial cells, most studies, with some notable exceptions, point toward a positive relationship between nanoparticle rigidity and cellular uptake;^8,9^ for cancer cells, on the other hand, the results are mixed.^9,10^ Thus, the interplay between multivalancy and nanoparticle rigidity for cellular internalization of biological nanoparticles is still open for research.

In the field of drug and vaccine delivery, liposomes, i.e., highly customizable small lipid vesicles, are of particular interest as efficient carriers for a vast variety of biologically active molecules.^2,3^ Their mechanical properties are determined by the lipid bilayer stretching and bending rigidities,^11^*k*_s_ and *k*_b_. In fact, a lipid bilayer is very tough with respect to stretching, and the liposome surface area can be considered constant. This means that the lipid bilayer stretching can be neglected and that the energy of liposome deformation can be identified primarily with the bending energy (which depends on lipid composition) and with osmotic pressure.^12^

Experimentally, the mechanical properties of lipid bilayers are traditionally quantified by using methods such as thermal fluctuation spectroscopy or by mechanical manipulation with optical tweezers.^13^ These methods are, however, typically applied to giant unilamellar vesicles and bilayer stacks and are not suitable to quantify ∼100 nm diameter liposomes. This difference in sizes is important, because there are indications that the bending rigidity, *k*_b_, appreciably increases with decreasing diameter down to ∼100 nm.^14^ More recently, atomic force microscopy (AFM) has become popular for characterizing surface-bound nanoscale liposomes through their controlled deformation using tip induced indentation.^15−18^ In particular, the attachment of ∼100 nm diameter biotin-modified liposomes to a streptavidin-modified supported lipid bilayer (SLB) was scrutinized.^18^ The corresponding results differ, however, depending on the mathematical model used. Other complications are related to limited statistics provided by AFM.

Vesicle deformation induced upon adsorption on a solid support has also been studied by tracking the effect of osmotic pressure on the quartz crystal microbalance signal,^12^ by using dual-wavelength measurement with a multiparametric surface plasmon resonance (MP-SPR),^19^ or by employing localized surface plasmon resonance (LSPR).^20^ The interplay between liposome binding and ligand–receptor valency has been previously investigated with QCM-D (with emphasis on ∼100 nm diameter unilamellar vesicles) and fluorescence microscopy (with emphasis on giant unilamellar vesicles), demonstrating that, as expected, the shape of biotin-modified liposomes to a streptavidin-modified supported lipid bilayer (SLB) depends not only on the streptavidin receptor concentration in the SLB but also on the biotin ligand concentration in the liposome^21^ due to redistribution of biotin in the liposomes and SLB (see, e.g., the model calculations^22^). Recently, a similar system was explored with the use of LSPR, suggesting that at an appreciable liposome biotin ligand concentration (>1%) the shape of 100 nm biotin-modified liposomes depends on this concentration,^14^ which was used to estimate the bending modulus. In fact, the studies in this area are just beginning, and there are so far only a few studies focusing on biotinylated liposomes (∼100 nm diameter liposomes^18,21^ and giant vesicles^21^), and many aspects concerning specific systems remain elusive.

In this SPR-based work, the binding of biotinylated liposomes to a streptavidin-functionalized SLB is investigated to gain further insights into this interplay by introducing liposome rigidity as an additional measurement parameter. Traditionally, SPR is used to analyze biomolecular interaction kinetics by measuring the shift in either wavelength or angle of the SPR minimum related to changes in the interfacial refractive index within an exponentially decaying field near (∼100 to 200 nm) the surface. These data are employed to offer reliable information about biomolecular surface coverage by using known relations between the biomolecular refractive index and molecular mass.^23,24^ Additional information is gained when SPR is operated at multiple wavelengths since the interface then can be probed with different decay lengths, which in turn enables the film thicknesses or average height of surface-adsorbed nanoscopic particles to be determined, assuming the mass to be uniformly distributed along the vertical coordinate.^19^ With suitable extension of the mathematical basis, this technique allows one to operate not only with average thickness or sizes but also to quantify the shape of nanoparticles, i.e., to take the nonuniform mass distribution into account.^19,24^

Here, we have explored these features of dual-wavelength SPR as a means to quantify the deformation of liposomes of different membrane compositions (in gel and fluid phases) bound to different types of supported cell membrane mimics via ligand–receptor (biotin–streptavidin) pairs. The results are compared with direct liposome adsorption on a solid SiO_2_ surface, where the deformation of fluid-phase liposomes is sufficiently strong to induce the vesicle collapse and formation of a planar supported lipid bilayer, while gel-phase liposomes remain unruptured but significantly deformed. A brief comparison with earlier studies^14,18,21^ focused on attachment of biotinylated liposomes to the SLB is given as well.

## Experimental Section

### Materials

1,2-Dioleoyl-*sn*-glycero-3-phosphocholine (DOPC; MW 786.1 Da), 1,2-distearoyl-*sn*-glycero-3-phosphocholine (DSPC; MW 790.1), 1,2-dioleoyl-*sn*-glycero-3-phosphoethanolamine-*N*-(cap biotinyl) (DOPE-cap biotin; MW 1105.5 Da), 1,2-distearoyl-*sn*-glycero-3-phosphoethanolamine-*N*-[biotinyl(polyethylene glycol)-2000] (DSPE-PEG(2000)-biotin; MW 3016.8 Da), and 1-palmitoyl-2-oleoyl-*sn*-glycero-3-phosphocholine (POPC; MW 760.1 Da) were purchased from Avanti Polar Lipids Inc. (USA). Streptavidin (SA) and glycerol were purchased from Sigma-Aldrich (Germany).

### Liposome Preparation

Liposomes made to form supported lipid bilayers (SLBs) as membrane mimics were composed of POPC and 0.5, 3, or 5 mol % DOPE-cap biotin and were produced with the use of the lipid film rehydration and extrusion method.^25^ Lipid solutions in 1:1 v/v chloroform/methanol were dried by rotary evaporation under reduced pressure (200 mbar) into thin films in round-bottom flasks. Trace amounts of solvent were removed by vacuum overnight. The films were dissolved by gentle vortexing to 8 mM lipid concentration in NaAc–NaCl buffer (10 mM sodium acetate, 150 mM NaCl, pH 5.0). The suspension was further diluted to a final lipid concentration of 4 mM and then extruded 21 times through a 100 nm nucleopore track-etched polycarbonate membrane (Whatman, U.K.), by use of a miniextruder (Avanti Polar Lipids Inc., USA) and at 1 bar pressure.

Fluid- and gel-phase liposomes (denoted DOPC-PEG-biotin and DSPC-PEG-biotin, respectively) comprised either DOPC or DSPC as the main lipid and 0.54 or 0.37 mol % DSPE-PEG(2000)-biotin, respectively. The difference in DSPE-PEG(2000)-biotin content aimed to achieve similar ligand densities, considering that gel-phase lipids pack more densely than fluid-phase ones; DSPC lipids have footprints of 0.497 nm^2^ and DOPC lipids have footprints of 0.725 nm^2^.^26,27^ DOPC-PEG-biotin liposomes were prepared as described above, with the addition of 10 freeze–thawing cycles of the 8 mM suspension prior to dilution and extrusion, in liquid nitrogen and a 50 °C water bath. The lipid mixture for DSPC-PEG-biotin liposomes was dried under a nitrogen stream, followed by vacuum overnight. The film was dissolved to an 8 mM lipid concentration by 10 freeze–thawing cycles in liquid nitrogen and 65 °C water bath. The suspension was then diluted to 4 mM and extruded as previously described but at 65 °C.

### Nanoparticle Tracking Analysis

The size distributions and approximate particle numbers of the DOPC-PEG-biotin and the DSPC-PEG-biotin liposomes were determined with nanoparticle tracking analysis (NTA). For this, a Nanosight LM10 (Malvern, U.K.), equipped with a Hamamatsu C11440-50B/A11893-02 camera and a 488 nm laser, was used. Each measurement consisted of at least five movies, each 60 s long. Analysis was done with the NTA software, version 3.3, using camera level 11 and detection threshold 2, with finite track length adjustment disabled.

**Figure 1:**
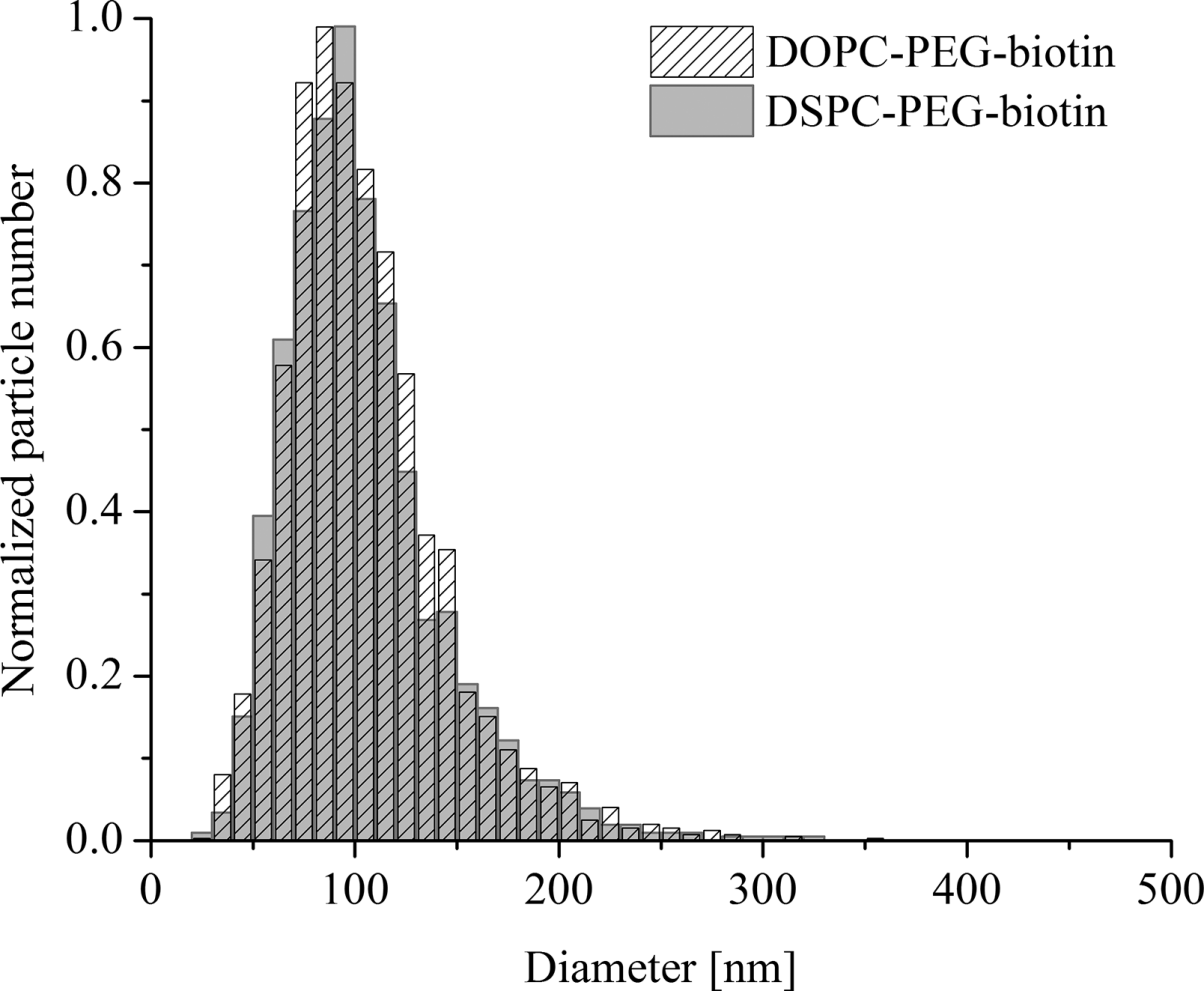
Size distribution (hydrodynamic diameter) of soft DOPC-PEG-biotin and rigid DSPC-PEG-biotin liposomes, determined with nanoparticle tracking analysis (NTA).

The size distributions of the two liposome types in solution, as determined with NTA at room temperature, were very similar, with both having a mean hydrodynamic diameter just over 100 nm (Figure 1).

### Multiparametric Surface Plasmon Resonance (MP-SPR)

Dual-wavelength SPR measurements were performed with the use of an MP-SPR Navi 220A NAALI (BioNavis, Finland), at 25 °C on SPR sensors with silica-coated gold plasmon surfaces. Before each measurement, the sensor was immersed in 10 mM sodium dodecyl sulfate solution for at least 1 h, thoroughly rinsed with Milli-Q water, dried with nitrogen, and UV-ozone treated for 20 min. SPR was monitored at wavelengths 670 and 785 nm, between 40 and 78°, where the angle of SPR minimum was observed and used as the measurement response. Glycerol (5 wt % in Milli-Q water) was injected at the beginning of each measurement, and the reversible shift in SPR response, along with the response following bilayer formation, was used for calibration purposes. SLBs with 0.5, 3, or 5 mol % biotin were formed by injection of the corresponding liposomes at an 80 μM lipid concentration, in PBS buffer (137 mM NaCl, 2.7 mM KCl, and 10 mM phosphate buffer solution, pH 7.4). SA was injected at 40 μg/mL (in PBS) and was followed by a PBS rinsing step. DOPC-PEG-biotin and DSPC-PEG-biotin were injected at 0.5 × 10^12^ particles/mL (diluted in PBS). The flow speed used was 10 μL/min, except for SA injection, where the flow speed was 20 μL/min. Measurements were repeated three times.

## Results and Discussion

To investigate how liposome rigidity affects interactions with receptors on a laterally mobile supported lipid bilayer (SLB), streptavidin-functionalized POPC SLBs formed on silica were subjected to biotin-modified fluid-phase (DOPC) and gel-phase (DSPC) liposomes and analyzed with MP-SPR. To facilitate SLB formation, the SLB contained DOPE-cap biotin, while the liposomes contained DSPE-PEG(2000)-biotin, which is often used for liposomes designed for drug delivery purposes. MP-SPR suits these investigations well, since both receptor and liposome coverage, as well as potential liposome deformation upon receptor interaction, can be quantified, thereby providing information on both the mass and the dimension of surface-bound entities.

### Determination of Bound Mass Using Dual-Wavelength SPR Measurements

**Figure 2:**
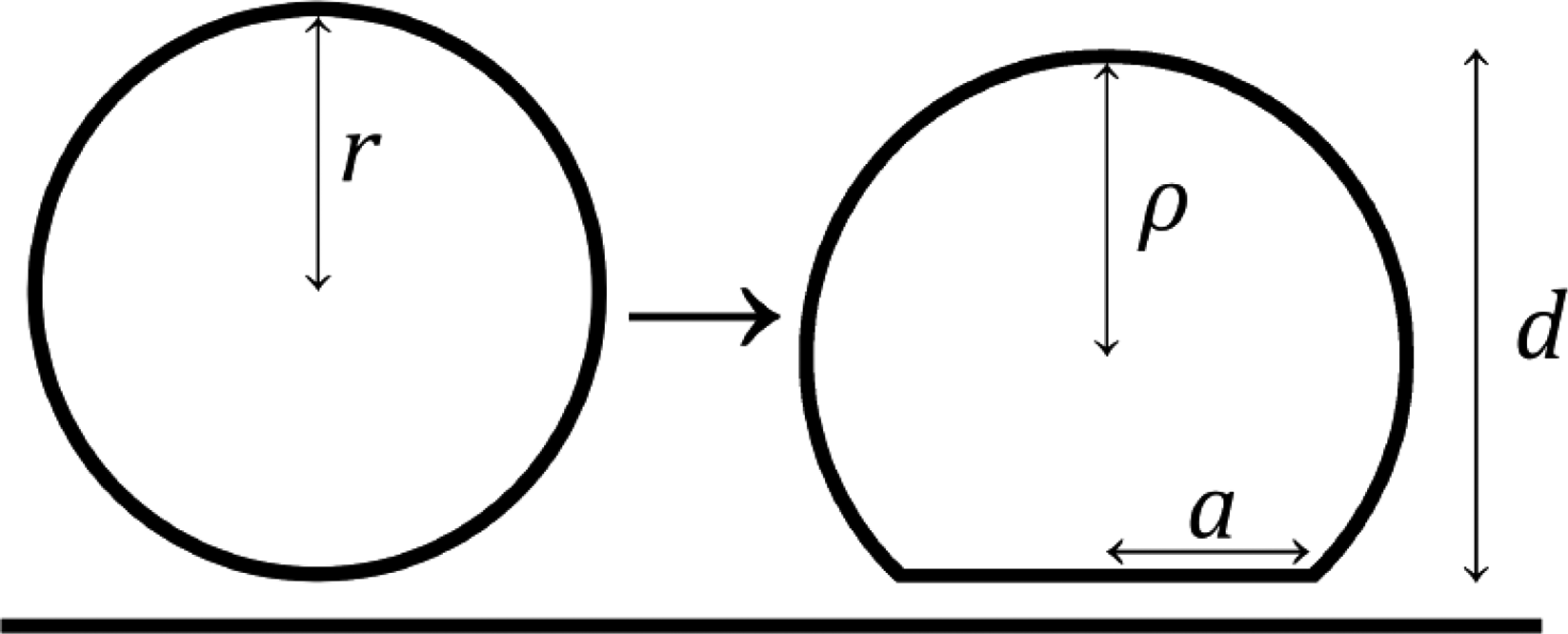
Liposome deformation is modeled as a sphere of radius *r* transitioning to a truncated sphere of radius ρ with a basement of radius *a* while the particle area is preserved. Prior to deformation, the liposome vertical dimension is simply 2*r*, while it after deformation equals *d*.

Our analysis of the liposome-related SPR signals is based on the results earlier obtained in ref (19). In particular, we recall that in general the SPR signal can be represented as
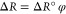
1where Δ*R*° is the signal calculated in the limit when the light-penetration depth, δ, is much larger than the size of adsorbed nanoparticles (Δ*R*° is proportional to the mass of adsorbed nanoparticles per unit area) and φ is a dimensionless factor (≤1) taking into account that the particle size may be comparable to, or larger than, δ. Using eq 1 with the conventional expression for Δ*R*°, the ratio of the signals measured at two wavelengths, λ_1_ and λ_2_, can be expressed as (eq 15 in ref (19))
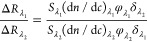
2where *S*_λ_ is the sensitivity factor and (d*n*/d*c*)_λ_ is the derivative of the refractive index with respect to the molecular concentration. In our case, the attached vesicles can be represented by a sphere in the undeformed state or by a truncated sphere with a flat basement (Figure 2) in the deformed state (this model was earlier used in the SPR context^19^ and also in the LSPR context^14^), and we have (see eq S1 in ref (19) and note that we have corrected a misprint there)
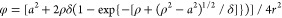
3where *r* is the liposome radius in the intact state, ρ = (4*r*^2^ + *d*^2^)/4*d* is the liposome radius in the deformed state (*d* is the vesicle height), and *a* is the corresponding vesicle support contact radius (Figure 2). Using eq 3, we take into account that, as mentioned in the Introduction, lipid bilayers are very tough with respect to stretching^28^ and accordingly consider that the liposome surface area is preserved upon deformation. Note also that eq 3 implies a uniform mass distribution in the liposomes. In our case, liposomes contain biotin, which can be redistributed nonuniformly upon liposome attachment to the SLB. The biotin mass per vesicle is, however, much smaller than the lipid mass, and accordingly the contribution of biotin to the SPR signal can be neglected.

The refractive index increment of ∂*n*/∂*c* is known for most types of biomolecules^29^ (0.146 and 0.138 mL/g^30^ for fluid- and gel-phase liposomes, respectively, and 0.185 mL/g for streptavidin), *S*_λ_1__ and *S*_λ_2__ can be estimated by calibration measurements of Δ*R* upon changing the bulk refractive index, Δ*n*_bulk_, at the respective wavelengths (Δ*R*_*λ*_ = *S*_*λ*_Δ*n*_bulk_), and the wavelength dependence of δ can be obtained theoretically.^31^ For pure gold sensors, we previously measured^19^*S*_λ_ and δ_λ_ and showed good agreement with theoretically determined decay lengths of δ_λ_1__ = 109 nm and δ_λ_2__ = 154 nm. However, since the sensors used in this work are coated with a 10–20 nm silica coating to enable SLB formation, the *S*_λ_ factors have to be adjusted to account for this difference in experimental parameters. This can be done by measuring the SPR signals during the SLB formation. In this case, we have *d* ≪ δ and eq 2 can be approximated as
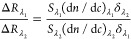
4Since (d*n*/d*c*)_λ_ and δ_λ_ are known and since SLBs fulfill the thin film approximation (*d* ∼ 5 nm), each sensor was calibrated based on the Δ*R*_λ_1__ and Δ*R*_λ_2__ responses upon SLB formation (see below), resulting in an estimated reduction of the (sensor specific) *S*_λ_1__/*S*_λ_2__ ratio of ∼1%. In fact, this correction is nearly negligible.

**Figure 3:**
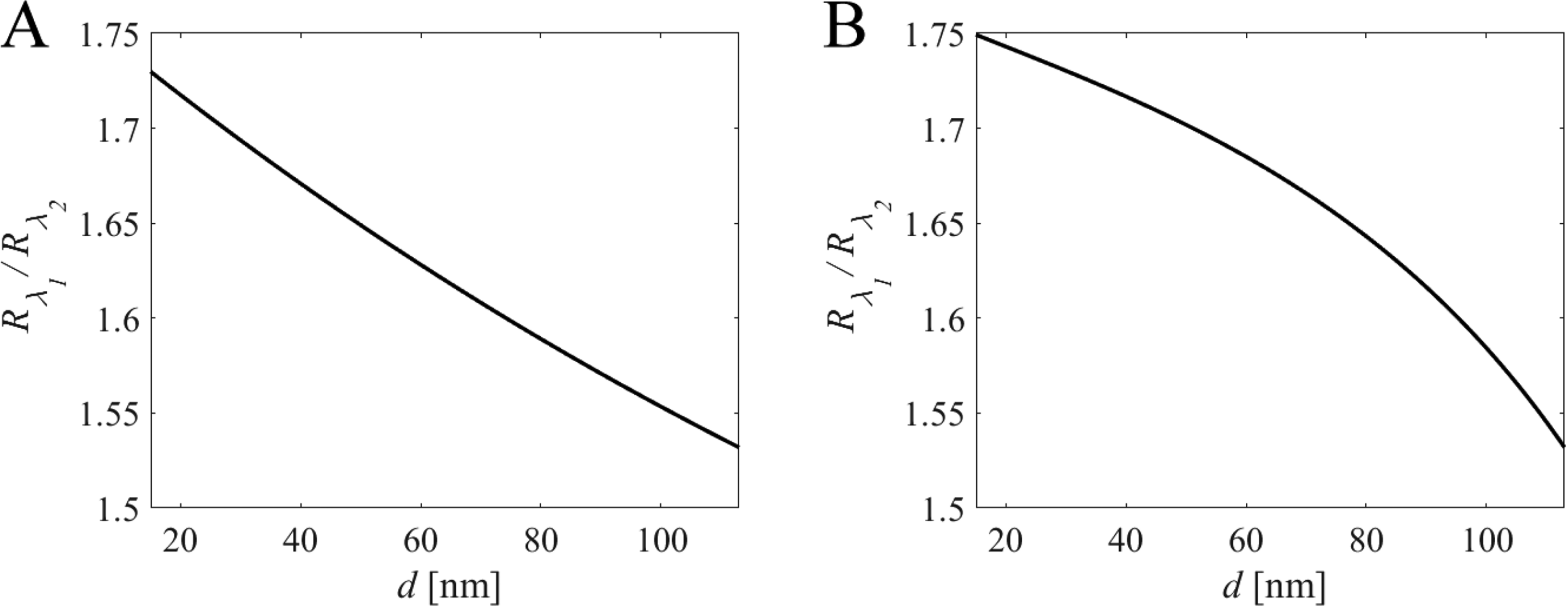
Ratio of SPR responses, *R*_λ_1__/*R*_λ_2__, with λ_1_ and λ_2_ of 670 and 785 nm, respectively, plotted versus liposome height, *d*, according to eqs 2 and 3 for (A) undeformed and (B) 105 nm size liposomes in the deformed state, and representative parameters for the silica-coated MP-SPR chips used in this study.

These parameters were subsequently employed to generate reference curves (Figure 3) by using eqs 2 and 3, from which the height, *d*, of vesicles was determined.

### Liposome Binding Data

**Figure 4:**
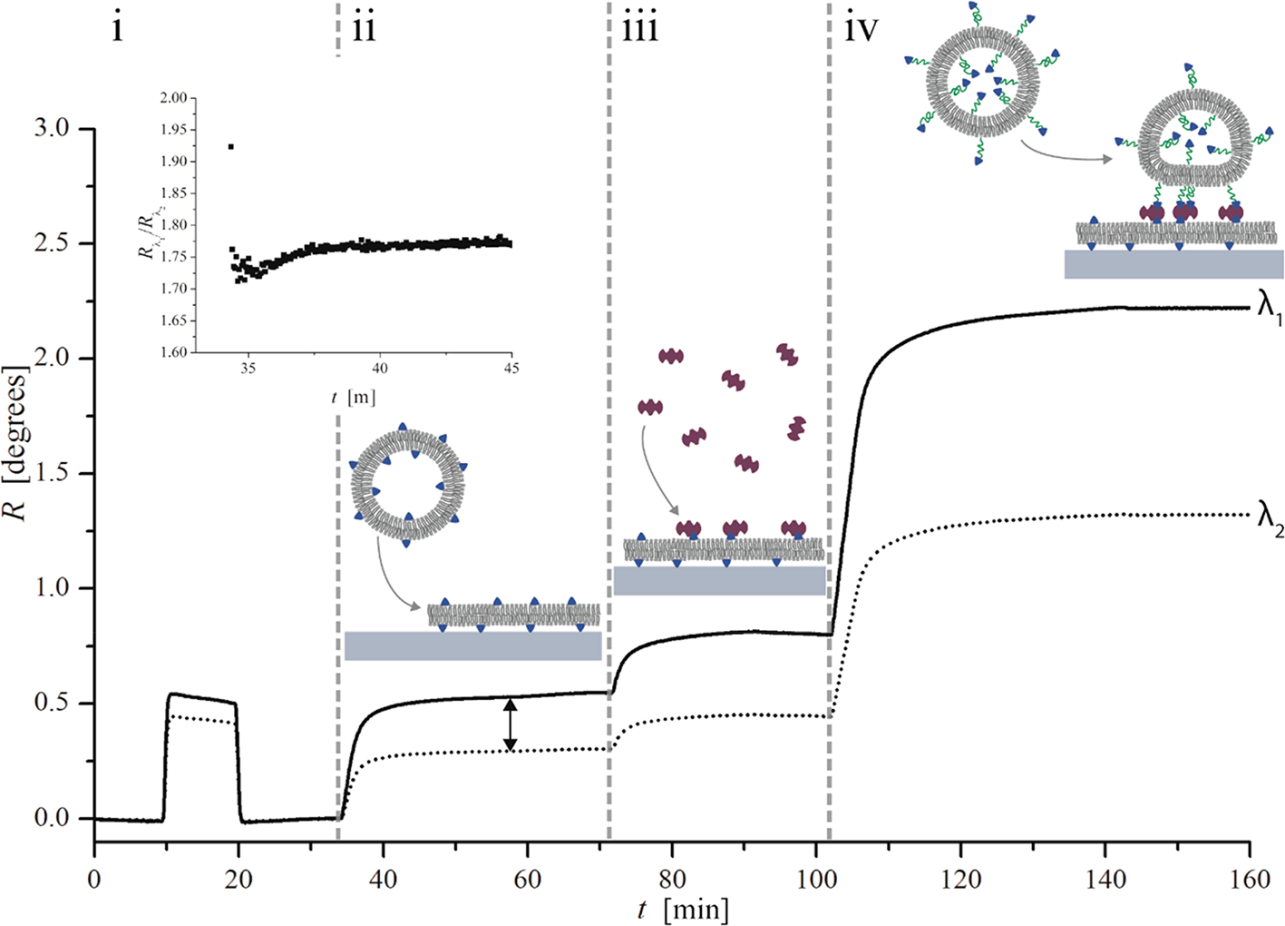
Illustration of a typical dual-wavelength SPR measurement. The angle of the SPR minimum is observed with (i) injection of 5 wt % glycerol for verification of the sensitivity constants, (ii) injection of liposomes and formation of an SLB containing 5 mol % DOPE-cap biotin (ΔΓ ∼ 345 ng/cm^2^), (iii) binding of SA (ΔΓ ∼ 120 ng/cm^2^), and (iv) binding of DOPC-PEG-biotin (ΔΓ ∼ 1100 ng/cm^2^, *d* ∼ 77 nm). The inset shows the *R*_λ_1__/*R*_λ_2__ ratio upon SLB formation plotted versus time used to calibrate the decay lengths.

A representative liposome binding experiment is shown in Figure 4, starting with the injection of 5 wt % glycerol for initial verification of the sensitivity constants *S*_λ_1__ and *S*_λ_2__; the bulk refractive index of the 5 wt % glycerol solution is obtained from the simultaneously measured shift in total internal reflection (TIR) angle obtained from the MP-SPR data, as described previously.^32^ Thereafter, addition of POPC-based liposomes was used to form an SLB containing 5 mol % DOPE-cap biotin (the SPR response acquired in this step was used to adjust the *S*_λ_ factors by ∼1% as discussed above), followed by addition of streptavidin, and, finally, addition of biotin-modified liposomes (in this example fluid-phase DOPC containing 0.54 mol % PEG-biotin lipids which translates to ∼480 PEG-biotin per liposome). The bound mass obtained using the procedure described above is for each step indicated in the legend of Figure 4.

**Figure 5:**
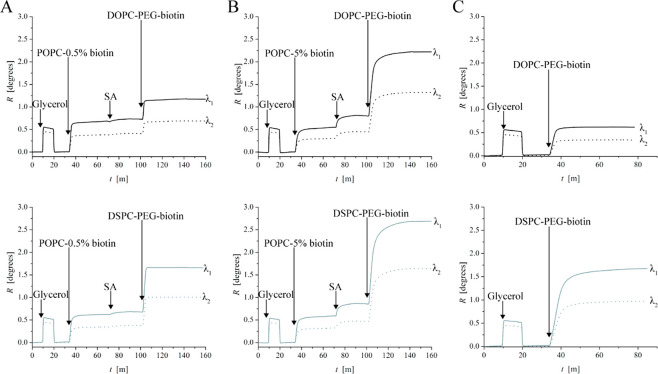
Dual-wavelength SPR sensograms for subsequent injections of (A) 5 wt % glycerol, POPC-0.5 mol % DOPE-cap biotin (forming an SLB), streptavidin (SA), and DOPC-PEG-biotin (top) or DSPC-PEG-biotin (below); (B) 5 wt % glycerol, POPC-5 mol % cap biotin (forming an SLB), streptavidin (SA), and DOPC-PEG-biotin (top) or DSPC-PEG-biotin (below); and (C) 5 wt % glycerol and DOPC-PEG-biotin (top) or DSPC-PEG-biotin (below) directly on silica.

The fluid-phase DOPC liposomes and gel-phase DSPC liposomes (made to contain an average of ∼480 PEG-biotin lipids per liposome (0.54 and 0.37 mol % PEG-lipids, respectively) used in this study were bound to SLBs with different coverages of streptavidin and analyzed in terms of their surface coverage and deformation as a function of SLB receptor concentration. The different streptavidin coverages on the SLBs were obtained by using compositions with either 0.5, 3, or 5% DOPE-cap biotin (subjected to a streptavidin solution until saturated binding). Figure 5 shows representative SPR responses for these measurements as well as reference data for the direct binding of the liposomes to silica-coated SPR sensors.

### Estimation of Streptavidin and Liposome Coverage

The mass of SLBs on the different sensor chips was on average ∼345 ng/cm^2^, which is in good agreement with previous mass uptake estimates using SPR.^33,34^ The surface coverage of streptavidin at 0.5 and 5% DOPE-cap biotin was ∼28 and 120 ng/cm^2^, respectively, which with a molecular weight of 60 kDa for streptavidin converts to protein coverages of ∼2.8 × 10^3^ and 12 × 10^3^ μm^–2^, respectively. The mass uptake of streptavidin on a 3% DOPE-cap biotin SLB was essentially the same as that for the 5% DOPE-cap biotin case, which shows that this coverage corresponds to the upper limit of streptavidin binding for this assay. A summary of the measured streptavidin coverage values and other main results of this study can be found in Table 1.

**Table 1:** Summary of Parameters of the Investigated SLB–Liposome Systems as Interpreted from the Measured Dual-Wavelength SPR Data^a^

	DOPC-PEG-biotin on 0.5% biotin SLB	DOPC-PEG-biotin on 5% biotin SLB	DSPC-PEG-biotin on 0.5% biotin SLB	DSPC-PEG-biotin on 5% biotin SLB
SA coverage	2.8 × 10^3^ μm^–2^	12 × 10^3^ μm^–2^	2.8 × 10^3^ μm^–2^	12 × 10^3^ μm^–2^
liposome coverage	28 μm^–2^	63 μm^–2^	50 μm^–2^	82 μm^–2^
liposome height after binding	90 nm	75 nm	105 nm	93 nm
contact area	4595 nm^2^	8370 nm^2^	805 nm^2^	3600 nm^2^
no. SA/liposome–SLB contact area	∼100	∼184	∼56	∼79

a The SA coverage and liposome coverage values are based on the SPR response interpreted as mass bound to the sensor surface during sample injection. The liposome heights after binding values, i.e., the liposome vertical dimensions, are based on the SPR response ratio for the two observed wavelengths, according to eqs 2 and 3 (the heights prior to binding are ∼105 nm). The contact area values are based on the measured liposome height combined with geometrical considerations according to eq 6 or, in the case of DSPC-PEG-biotin on 0.5% biotin bilayer, eq 7. Note that these contact area values assume no wrapping of the SLB around the liposomes. The no. SA/liposome–SLB contact area values are for the 0.5% biotin SLB cases, in which case all SA molecules are engaged in the liposome binding, estimated from the ratio between the SA and liposome coverages. Since not all SA molecules are engaged in liposome binding in the 5% biotin SLB cases, it is instead based on the measured contact area with a local SA coverage assumed to be equal to the measured SA coverage in the case of DOPC-PEG-biotin liposomes at 0.5% DOPE-cap biotin-SLB, i.e. ∼22 × 10^3^ μm^–2^.

In order to interpret the SPR response upon liposome binding to the streptavidin-coated SLBs in terms of number of bound entities per unit area, values for the mass per vesicle need to be estimated. These values were obtained from the lipid molar mass (see the Experimental Section) and the number of lipids per respective liposome (*N*_lipids_), approximated using geometrical considerations as follows:
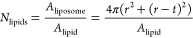
5where *A*_liposome_ is the surface area of a liposome with outer radius *r* and bilayer thickness *t*; *A*_lipid_ is the footprint of a single lipid. Using *A*_DOPC_ = 0.725 nm^26^ and *A*_DSPC_ = 0.497 nm^27^ and bilayer thicknesses of 3.7 and 4.8 nm for DOPC and DSPC, respectively,^26,27^ the mass values for a fluid-phase liposome and a gel-phase liposome with a diameter of 105 nm become 1.18 × 10^–7^ and 1.69 × 10^–7^ ng, respectively.

Considering first liposome binding to SLBs containing a low amount, 0.5 mol %, of DOPE-cap biotin, the mass uptakes of fluid- and gel-phase liposomes were ∼330 and 840 ng/cm^2^, respectively, which translate to coverages of 28 and 50 liposomes μm^–2^, respectively. The DLVO-type (Derjaguin–Landau–Verwey–Overbeek theory) interaction between adsorbed vesicles takes place on a length scale much shorter than their size^35^ and they are usually immobile, and accordingly, their maximum coverage can be estimated by using the conventional model of random sequential adsorption as ∼54%, which for liposomes with a radius of 52.5 nm as used in this work (Figure 1) corresponds to ∼62 liposomes μm^–2^. This means that the liposome coverage is lower than the jamming limit upon irreversible adsorption of immobile spheres, suggesting that in both cases (DOPC-PEG-biotin and DSPC-PEG-biotin liposomes), all available streptavidin molecules are engaged in liposome binding. The fact that the coverage of streptavidin in terms of bound entities per surface area is significantly higher than that of liposomes (∼2.8 × 10^3^ μm^–2^ versus 28 and 50 μm^–2^) and the appreciable value of the biotin–streptavidin interaction suggest that the fluid nature of the SLB leads to accumulation (harvesting) of all streptavidin molecules into the contact area between the liposome and the SLB surface. The number of streptavidin molecules accumulated in the contact zone of each bound liposome would then correspond to ∼100 and 56 streptavidin molecules for DOPC-PEG-biotin and DSPC-PEG-biotin, respectively. Assuming close packing of streptavidin (and an area of ≃29 nm^2^ per streptavidin molecule^36^), these numbers correspond to contact areas of ∼2900 and 1624 nm^2^, respectively.

**Figure 6:**
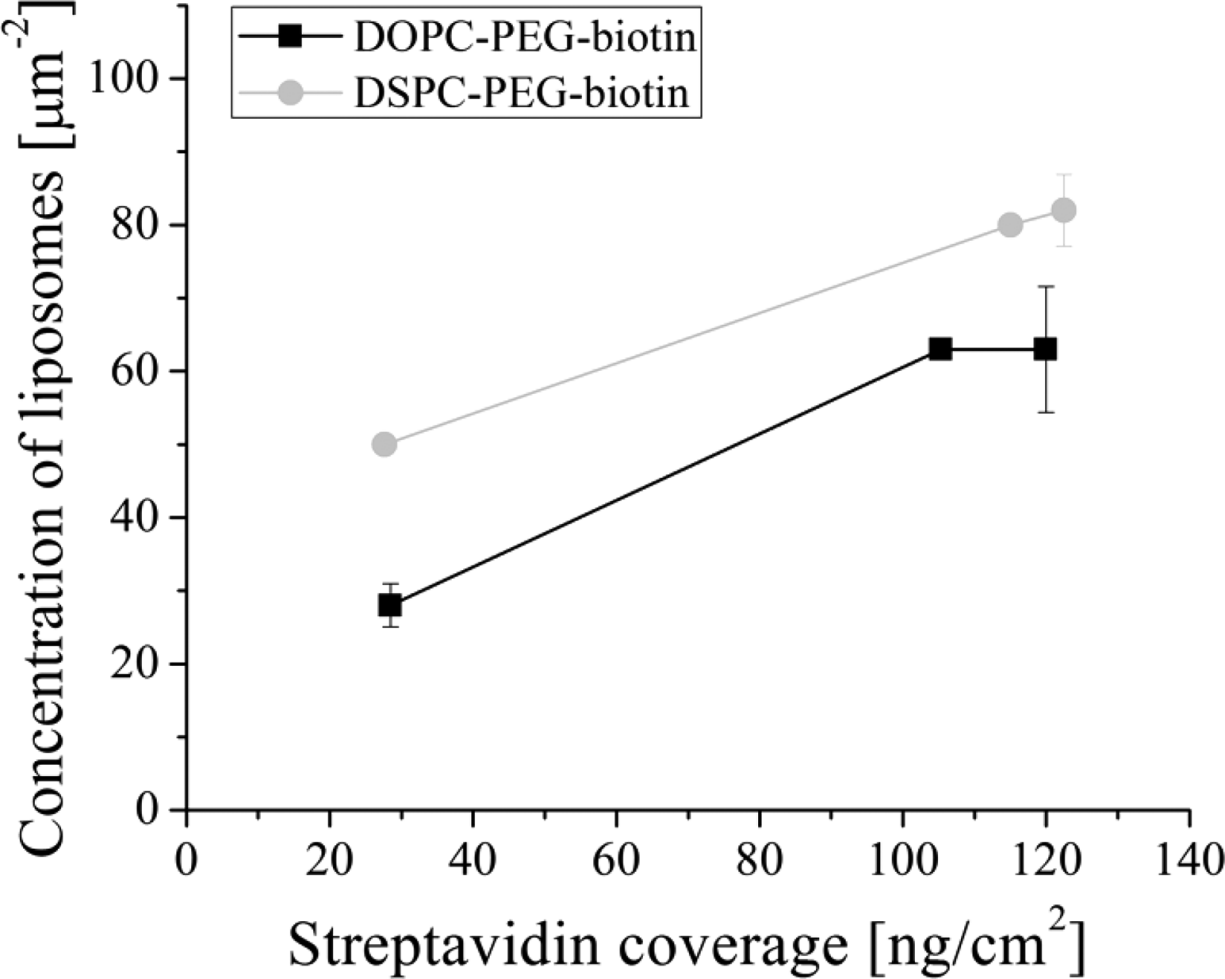
Surface concentration of liposomes per square micrometer as a function of streptavidin coverage on the SLB. Error bars show standard deviation.

Considering liposome binding to SLBs containing a relatively high amount, 5 mol %, of DOPE-cap biotin, and streptavidin coverage of ∼12 × 10^3^ μm^–2^, the mass uptakes of fluid- and gel-phase liposomes were ∼740 and 1390 ng/cm^2^, respectively, which convert to liposome coverages of 63 and 82 liposomes μm^–2^ (Figure 6). Liposome coverages close to, or higher than, the jamming limit of 62 μm^–2^, and higher than the coverage reached upon DSPC-PEG-biotin adsorption directly on highly adhesive SiO_2_ (Figure 5C), suggest that (i) liposome binding is, in this regime, limited not by the availability of streptavidin but rather by geometric constrains and/or (ii) the liposomes retain sufficient mobility for rearrangement into a more compact surface coverage than predicted by the jamming limit for random adsorption. In order to elucidate the viability of this interpretation, the contact area between the liposomes and the SLB needs to be compared to the streptavidin coverage. This, in turn, requires consideration of the liposome deformation upon binding.

### Analysis of Liposome Deformation

**Figure 7:**
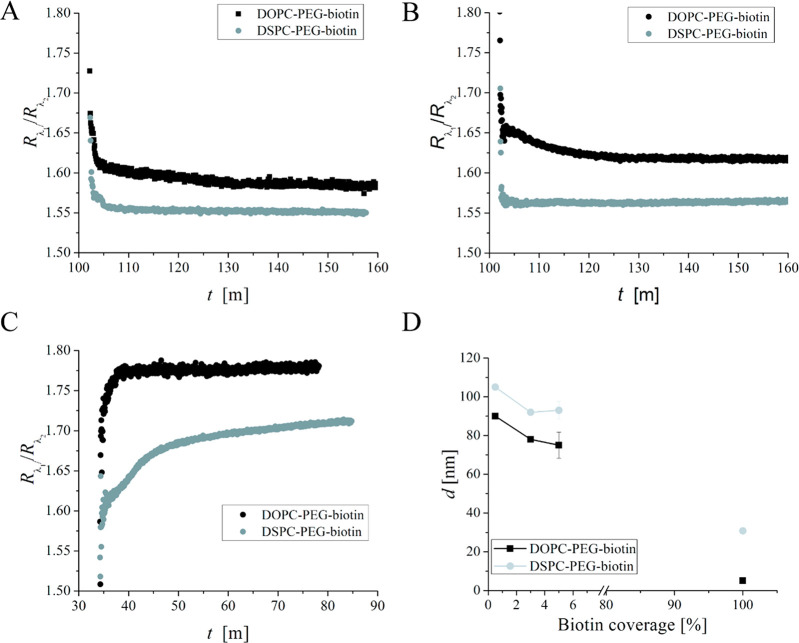
Plots of *R*_λ_1__/*R*_λ_2__ ratio versus time, visualizing DOPC-PEG-biotin and DSPC-PEG-biotin deformation upon binding to (A) SLB with 0.5 mol % cap biotin and low streptavidin coverage, (B) 5 mol % cap biotin and high streptavidin coverage, and (C) silica-coated MP-SPR sensor. (D) Film thickness/liposome height (*d*) versus biotin coverage, where 100% corresponds to adsorption directly on silica.

The liposome coverage values obtained above are based on the liposome height values as calculated from the *R*_λ_1__/*R*_λ_2__ ratios (Figure 7A–C) using eq 2. In addition, information about liposome height (deformation) can be used to calculate and compare contact areas between the liposome and the SLB and to compare these values with the available number of streptavidin molecules in the contact zone with the number of biotin ligands present on the liposomes. In the low streptavidin coverage regime (0.5 mol % DOPE-cap biotin SLB), the gel-phase (DSPC-PEG-biotin) liposome layer thickness was measured to be ∼105 nm, which is close to the liposome diameter of ∼105 nm as observed in NTA. This suggests that the gel-phase (DSPC-PEG-biotin) liposomes exhibit negligible deformation and that their measured height of 105 nm can serve as reference for the liposome diameter prior to deformation for both the DSPC-PEG-biotin and DOPC-PEG-biotin liposomes, since they were prepared to have identical diameters in suspension (Figure 1). The fluid-phase (DOPC-PEG-biotin) liposome height was measured to be 90 nm upon binding in the low streptavidin coverage regime (0.5 mol % DOPE-cap biotin SLB). These liposomes were thus deformed to this (average) value from their initial diameter of 105 nm according to the interpretation above. Under the assumption that the spherical liposomes adopt a truncated sphere geometry upon deformation, the area in contact with the underlying substrate, *A*_contact_, can be expressed as
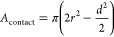
6

The fluid-phase DOPC-PEG-biotin liposomes that adhered to the low biotin coverage SLB were on average deformed from a spherical shape with a diameter of 105 nm to a height of 90 nm, which with the use of eq 6 converts to a contact area of 4595 nm^2^. With on average 100 streptavidin molecules being engaged in the binding of each DOPC-PEG-biotin liposome at 0.5% biotin-SLB (see above), the local streptavidin coverage in the contact zone corresponds to ∼22 × 10^3^ μm^–2^. This number is significantly higher than the maximum streptavidin coverage of ∼12 × 10^3^ μm^–2^ obtained on the flat SLBs (at 3 and 5% DOPE-biotin-cap), which supports that the binding involves a process of receptor harvesting into the contact zone between the liposomes and the SLB and that this, in the low-coverage regime, is the limiting factor for the extent of liposome binding and deformation. This streptavidin coverage agrees well with the value obtained for the theoretical jamming limit upon random sequential adsorption of immobile spheres (i.e., a model for streptavidin), which assumes a 54% maximum coverage.

To calculate the contact area between the nondeformed gel-phase DSPC-PEG-biotin liposomes and the underlying SLB, which, as discussed above, appear to contain ∼56 streptavidin molecules per liposome, one should recall that the liposomes were modified by means of biotin-modified PEG with a molecular weight of 2 kDa, which is expected to provide a flexible polymer brush surrounding the liposomes. At the concentrations used in this work, the PEG would assume a mushroom conformation extending ∼4 nm from the bilayer;^37^ however, high ligand density or external forces can almost double that length.^38^ If the PEG molecules at the edge of the contact zone between the SLB and the liposome are fully extended, the corresponding disk-shaped contact area between the SLB and the polymer brush can, from simple geometrical considerations, be expressed as
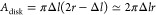
7where Δ*l* is the increase in the length of the PEG chain from its relaxed Flory radius to its fully extended form. Assuming a change in length of the PEG from 4 to 6.5 nm,^38^ this corresponds to a disk area of ∼805 nm^2^, which is 2% of the total liposome area, 4π(105/2)^2^ nm^2^. If we assume that the biotin ligands are immobile due to the gel-phase environment and are evenly distributed on the liposome surface, 2% of the ∼240 ligands per liposome corresponds to just ∼5 PEG-biotin in the contact zone, an unreasonably low number considering the measured 56 SA molecules bound. This suggests that the ligands on DSPC-PEG-biotin are indeed mobile on these short length scales, or unevenly distributed. Alternatively, the underlying bilayer undergoes restructuring and partial encapsulation of the rigid liposomes, which was previously observed for streptavidin-modified SLBs upon binding of gold nanoparticles modified with PEG-biotin.^39^

Turning to the high streptavidin (and liposome) coverage regime, contraction from ∼105 nm to heights of 75 and 93 nm was observed for fluid- and gel-phase liposomes, respectively (Figure 7D), which for truncated sphere geometries convert (using eq 7) to contact areas of 8370 and 3600 nm^2^, respectively. Under the assumption that the local coverage in the contact zone can reach that estimated for the case of DOPC-PEG-biotin liposomes at 0.5% DOPE-cap biotin-SLB streptavidin coverage, i.e., ∼22 × 10^3^ μm^–2^, this corresponds to ∼184 and ∼79 streptavidin molecules being engaged for binding of a DOPC-PEG-biotin liposome and a DSPC-PEG-biotin liposome, respectively. It should further be noted that, for the DOPC-PEG-biotin case, the total liposome coverage (∼63 μm^–2^) multiplied by the number of streptavidin molecules in the contact zone (∼184) is fairly close to the total number of available streptavidin molecules (∼12 × 10^3^ μm^–2^), suggesting that, similarly to the 0.5% biotin-SLB case, the degree of deformation is limited by the streptavidin available to harvest into the contact zone. In contrast, the deformation of DSPC-PEG-biotin appears to be limited by the stiffness of the liposome membrane. It cannot be excluded, though, that the overall structural changes observed also involve SLB restructuring and liposome encapsulation. That phenomenon has previously been proposed to explain clustering of norovirus-like particles upon binding to glycosphingolipid receptor-modified SLBs.^40^ This interpretation is further supported by the liposome deformation observed upon adsorption of both liposome types to silica, in which case the lipid–surface interaction, rather than the ligand–receptor interaction, controls the adhesion strength. On silica, the gel-phase liposomes deform to a film thickness of *d* ≈ 30 nm, while for fluid-phase liposomes the interaction is sufficiently strong to cause liposome collapse and SLB formation (Figure 7D). The observed degree of deformation for the gel-phase liposomes is somewhat larger than that recently obtained for similar liposomes using AFM,^41^ which is likely attributed to slight differences in vesicle preparation, surface cleaning, and buffer conditions. Hence, it appears likely that on the SLB, which served to mimic a cellular membrane, deformation of fluid-phase liposomes is limited by the number of available ligand–receptor bonds while membrane stiffness seems to play a significant role in limiting the deformation of gel-phase liposomes.

The higher than expected number of streptavidin molecules involved in DSPC-PEG-biotin suggests a heterogeneous distribution of the PEG-biotin ligands over the surface of the liposome. Indeed, phosphocholine-based gel-phase liposomes have previously been observed to have highly disordered grain boundaries between ordered gel-phase facets.^42^ Considering the difference in critical packing parameter (lipid geometry) between DSPC and DSPE-PEG(2000)-biotin, it is not unlikely that the PEGylated lipids would cluster in the disordered boundary regions, giving rise to high local ligand concentrations. It is noteworthy that such clustering, in either the grain boundaries of gel-phase liposomes or in the contact zone underneath, could cause local enrichment of PEG-lipids to an extent that induces the conformational transition from mushroom to brush conformation. Thus, the thickness of the PEG layer is a point of uncertainty, which may lead to overestimation of the liposome dimension judged from MP-SPR data.

To put these results in the context of previous work, it is worthwhile to note that recently, as already noted in the Introduction, similar studies aimed at fluid-phase liposomes have been performed by using other approaches including LSPR,^14^ AFM,^18^ and QCM-D.^21^ A brief summary of the results obtained in our study and in refs (14) and (21) is given in Table 2 (the results reported in ref (18) are not included due to its dissimilarities in liposome composition and surface chemistry). The first general conclusion from these data is that the deformation of liposomes is modest in all cases. Another conclusion is that the biotin concentration in the SLB seemingly plays a nearly negligible role for the deformation of the 70 nm liposomes,^14^ whereas its role in our study of the 105 nm liposomes is somewhat more appreciable. This difference may be related to the liposome coverage. In particular, the quantitative results for 70 nm liposomes^14^ were obtained primarily at appreciably lower coverages than those in our case.

**Table 2:** Compilation of Results Concerning the Deformation (Here Quantified as the Ratio *d*/2*r*, i.e., between the Liposome Vertical Dimension and the Initial Diameter) of Biotinylated Liposomes of Different Main Constituting Lipids, Phases, and Molar Fractions of Biotinylated Lipids, Attached to Streptavidin-Functionalized SLB of Different Molar Fractions of Biotinylated Lipids^a^

T	S	θ_1_	θ_SLB_	C	2*r* [nm]	*d*/2*r*	ref
DOPC	F	0.0054	0.005	S	105	0.857	b
DSPC	G	0.0037	0.005	S	105	1	b
DOPC	F	0.0054	0.05	S	105	0.714	b
DSPC	G	0.0037	0.05	S	105	0.886	b
DOPC	F	0.00125	0.01	I	70	0.970	(14)
DOPC	F	0.0025	0.01	I	70	0.896	(14)
DOPC	F	0.005	0.01	I	70	0.810	(14)
DOPC	F	0.01	0.01	I	70	0.795	(14)
DOPC	F	0.02	0.01	I	70	0.825	(14)
DOPC	F	0.01	0.00125	I	70	0.810	(14)
DOPC	F	0.01	0.0025	I	70	0.780	(14)
DOPC	F	0.01	0.005	I	70	0.745	(14)
DOPC	F	0.01	0.01	I	70	0.795	(14)
DOPC	F	0.01	0.02	I	70	0.754	(14)
DOPC	F	0.004	0.006	S	100	0.872^c^	(21)
DOPC	F	0.02	0.006	S	100	0.872^c^	(21)

a Main constituting lipids, T; phase, S; molar fraction of biotinylated lipids, θ_1_; streptavidin-functionalized SLB of different molar fractions of biotinylated lipids, θ_SLB_. The deformation was investigated in different kinetic phases of the liposome attachment C: S (close to saturation) or I (during the initial phase). The *d*/2*r* values provided here are based on the liposome–SLB contact area values supplied in the respective references and recalculated using eq 6.

b Results presented in this article.

c The liposome vertical thickness value *d* is based on the contact area value claimed in the reference, but in order to be consistent with the other table values, the contact area was recalculated by using a 29 nm^2^ area per streptavidin molecule instead of the 25 nm^2^ used in the reference.

Concerning more specific details, it is clear that the liposome deformation process is governed by multiple parameters, including (i) the number and strength of ligand–receptor bonds, (ii) the membrane bending constant, *k*_b_, (iii) osmotic pressure, and (iv) the size and composition influencing (i) and (ii). The interplay between these factors is important to consider. In general, the formation of the ligand–receptor pairs is energetically favorable and should eventually be counterposed by other factors. It is of interest that, despite a similar scale of deformation of gel-phase liposomes, the earlier conclusions concerning this factor are different and imply membrane bending^14^ with a high value of *k*_b_ (∼700*k*_*B*_*T*) and high adhesion-induced osmotic pressure (∼0.5 MPa) in combination with *k*_b_ = 10–30*k*_B_*T* (Figure 4a in ref (18); note that the liposomes used in that work are not biotinylated). In our case, the number of biotin–streptavidin complexes is ∼56–184, the binding energy per complex is ∼12 kcal/mol,^14^ i.e., around 20*k*_*B*_*T*, and, accordingly, the total binding energy is on the order of 2 × 10^3^*k*_B_*T*. The osmotic pressure is determined primarily by NaCl, and its scale is *P*_os_ = 2Δ*ck*_B_*T*, where Δ*c* = 150 – 137 = 13 mM is the difference of the NaCl concentrations during the preparation of and experiments with liposomes. The deformation of liposomes is modest, and the corresponding scale of the change of the osmotic pressure related energy can be estimated as
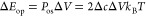
8where Δ*V* is the scale of the change of the liposome volume. With Δ*c* = 13 mM and Δ*V* = 0.1*V*_0_ = 5 × 10^–17^ cm^3^ (*V*_0_ is the liposome volume in the undeformed state), we have Δ*E*_op_ ∼ 800*k*_B_*T*. This energy is smaller that the above-estimated total binding energy by a factor of 3. This factor is not large, and accordingly we cannot exclude the osmotic pressure. The high value of the membrane bending constant, *k*_b_ ∼ 700*k*_B_*T*, cannot be excluded either.

## Conclusion

The dual-wavelength SPR approach presented in this work offers the possibility to investigate the interplay between affinity, avidity, and particle rigidity in the context of nanoparticle adhesion and deformation on supported lipid bilayers, providing results not easily obtained using alternative means. In this way, we successfully investigated the deformation of fluid- and gel-phase liposomes and how it relates to the valency of the interaction between liposomes and cell membrane mimics. It is worth pointing out that we varied the rigidity of the nanoparticles while keeping the ligand density constant at a number typical in the context of vaccine and drug delivery formulations.^43−45^ In many applications, literature data suggest that rigid nanoparticles are ideal for optimal cellular uptake.^9,10,46−48^ The same holds true for particles with high avidity,^49,50^ which suggests that, in future investigations, the ligand density should also be systematically varied and correlated with liposome deformation for different membrane compositions. In fact, systematic variation of the ligand density and liposome dimension, in combination with theoretical modeling, might enable determination of the bending rigidity of the lipid bilayer in small liposomes. This is possible provided that the binding energy of ligand–receptor pairs can be accurately determined, experimentally or theoretically. Vice versa, the binding energy can be estimated provided the bending rigidity is known. Thus, in the future, the dual-wavelength SPR approach has the potential to be used to address these questions for a variety of different nanoparticles, including pharmaceutical carriers, as well as biological particles such as exosomes and viruses. Additionally, the possibilities to alter the nature of the supported cell-membrane mimic are virtually endless and offer opportunities to study interactions ranging from the specific and well-defined, e.g., particular receptor–ligand pairs of interest, to the more complex, but biologically more relevant, situation obtained utilizing native cell-derived SLBs.^51^

## Abbreviations

DOPC: 1,2-dioleoyl-*sn*-glycero-3-phosphocholine
DOPC-PEG-biotin: biotinylated DOPC-based liposomes
DSPC: 1,2-distearoyl-*sn*-glycero-3-phosphocholine
DSPC-PEG-biotin: biotinylated DSPC-based liposomes
DOPE-cap biotin: 1,2-dioleoyl-*sn*-glycero-3-phosphoethanolamine-*N*-(cap biotinyl)
DSPE-PEG(2000)-biotin: 1,2-distearoyl-*sn*-glycero-3-phosphoethanolamine-*N*-[biotinyl(polyethylene glycol)-2000]
MP-SPR: multiparametric surface plasmon resonance
NTA: nanoparticle tracking analysis
POPC: 1-palmitoyl-2-oleoyl-*sn*-glycero-3-phosphocholine
QCM-D: quartz crystal microbalance with dissipation monitoring
SA: streptavidin
